# Mapping non-response in a prevention program for cardiometabolic diseases in primary care: How to improve participation?

**DOI:** 10.1016/j.pmedr.2020.101092

**Published:** 2020-04-08

**Authors:** Ilse F. Badenbroek, Marcus M.J. Nielen, Monika Hollander, Daphne M. Stol, Astrid E. Drijkoningen, Roderik A. Kraaijenhagen, Niek J. de Wit, François G. Schellevis

**Affiliations:** a Julius Center, University Medical Center Utrecht, P.O. Box 85060, 3508 AB Utrecht, The Netherlands; b Netherlands Institute for Health Services Research (NIVEL), The Netherlands; c NDDO Institute for Prevention and Early Diagnostics (NIPED), Amsterdam, The Netherlands; d Department of General Practice & Elderly Care Medicine/EMGO Institute for Health and Care Research, VU University Medical Center, Amsterdam, The Netherlands

**Keywords:** Primary Prevention, Refusal to Participate, Risk Assessment, Health Risk Behaviors, Cardiovascular Diseases, Risk Factors, CMD, Cardiometabolic diseases, RS, risk score, BMI, Body mass index, CVD, Cardiovascular disease

## Abstract

Non-response in prevention programs for cardiometabolic diseases (CMD) in primary care is often overlooked. The aim for this study was to define factors that influence the primary response to a selective CMD prevention program and to determine response-enhancing strategies that influence the willingness to participate. We conducted a non-response analysis within a randomized controlled trial evaluating a selective CMD prevention program, the study was conducted from 2013 to 2018 in Netherlands. A random sample of 5616 patients from 15 general practices were invited to complete a risk score (RS) as initial step of the program. Non-responders received an additional questionnaire. The response on the risk score was 51% (n = 2872). From the 3558 non-response questionnaires sent, 786 (22%) were returned. In a multivariable multilevel regression analysis smoking was independently associated with non-response. Of all reported reasons for non-response ‘forgot/no time’ accounted for 45%. In total, 73% of the non-responders indicated to reconsider participation when approached differently. A personal approach by the patients’ own GP, using advertisements and informative campaigns are potentially the best methods to enhance the response. Although a relatively high proportion did not respond to the invitation for the risk score, the majority of them indicated to be willing to participate if a different invitation strategy would be used. With more time and energy, response rates for CMD prevention programs could possibly increase substantially. A next logical step in this process is to test potential response enhancing strategies in research setting.

## Highlights

• Non-response in prevention programs for CMD in primary care is often overlooked.
• Willingness to participate amongst non-responders is high.
• There are response enhancing strategies that show potential.
• We should be able to boost response rates of prevention programs for CMD.
• A next logical step is to test potential response enhancing strategies.

## 1 Introduction

Cardiometabolic diseases (CMD) including cardiovascular disease, diabetes mellitus and chronic kidney disease account for a large part of the disease burden and health care costs. The prevalence of CMD is bound to increase in the next decades due to an aging population with an unhealthy lifestyle (WHO, 2010). Most of the risk for CMD is attributable to modifiable risk factors such as smoking, unhealthy diet, obesity and physical inactivity: for example, 90% of the risk for an acute myocardial infarction is determined by these risk factors (Yusuf et al., 2004). This calls for preventive actions aimed at stimulating people to adopt a healthier lifestyle. Worldwide many different prevention and screening programs for CMD have been developed to suit this purpose (Cooney et al., 2009, Dyakova et al., 2016).

In order for these prevention programs to be successful and cost-effective, high participation rates are crucial (Cooney et al., 2009, Koopmans et al., 2012, Søgaard et al., 2013). Health effects on population level increase with rising participation and compliance rates: with 100% participation and full compliance to CMD prevention programs 93% of all cardiovascular deaths could be prevented (Cooney et al., 2009). However, participation rates in studies persistently show large variations in participation, ranging from 3% to 75% (Godefrooij et al., 2012, Van der Meer et al., 2013), but 100% participation and compliance seems unrealistic (Koopmans et al., 2012, Nielen et al., 2010). The NHS health checks that were introduced in the UK in 2009 were modeled at a participation rate of 75%, but even this rate has not been reached in most regions (Jenkinson et al. 2015).

Low participation rates are a major problem in the implementation of CMD screening and prevention programs in general. If factors that lead to non-participation in preventive strategies could be determined, it might reveal opportunities to address a large group that up to now has been out of reach. Several studies focused on the characteristics of non-responders in screening and prevention programs for CMD. Most of these studies found non-responders to be more often of younger age and to be a smoker (Cochrane et al., 2013, Dalton et al., 2011, Hoebel et al., 2014, Klijs et al., 2012, Lambert et al., 2012, Lang et al., 2016, Thorogood et al., 1993, Van Den Brekel-Dijkstra et al., 2016, Waller et al., 1990). Unfortunately, these studies did not provide a consistent profile of non-responders, nor did they lead to evidence based recommendations to increase participation rates.

Within the context of the large-scale INTEGRATE study, which focuses on the (cost-)effectiveness of a stepwise CMD prevention program in primary care (Badenbroek et al., 2014), we studied determinants of response to the first step of the prevention program, the self-reported risk score (RS). This response rate determines the domain of the follow-up steps and is vital for the overall success of the program. Therefore, we compared responders with non-responders, aiming to identify factors that influence response to the initial CMD risk score. Such factors can serve as a starting point for response-enhancing strategies that could improve participation rates.

## 2 Methods

### 2.1 INTEGRATE study

This cross-sectional study was performed within the framework of a trial, the INTEGRATE study. The INTEGRATE study is a stepped-wedge randomized controlled trial that was conducted in 2014 to 2017 in the Netherlands (Badenbroek et al., 2014). The aim of the INTEGRATE study is to assess the effectiveness and cost-effectiveness of a stepwise CMD prevention program coupled to an individualized lifestyle intervention. The detailed study design of the INTEGRATE study is described elsewhere (Badenbroek et al., 2014).

### 2.2 Study population

To ensure practicability we used a random sample of 15 of the 37 participating practices in the INTEGRATE study for the non-response analysis, with a total of 5616 eligible patients for the prevention program.

### 2.3 Steps of the INTEGRATE study

In the INTEGRATE study 37 general practices approached all patients between 45 and 70 years old without known CMD, hypertension or hypercholesterolemia. Eligible patients were randomized into an intervention and a waiting list control group. The patients in the waiting list control group received the intervention after one year as well. As the first step of the prevention program, patients received a personal letter from their GP with an invitation to assess their CMD risk through an online risk score (RS). After two weeks a reminder letter was sent to those who did not respond to the first invitation. The reminder invitation also contained a paper version of the RS and a returning envelope. Non-response questionnaires were sent to patients who did not respond online to the call for participation within four weeks after the first invitation. Non responders were identified based on a pseudonymized participation log that was kept by the study team. After filling in the RS, patients with an increased risk were advised to make an appointment with the GP for the second step of the prevention program to complete their CMD risk profile with additional measurements. Patients with a low risk for cardiometabolic diseases received online tailored lifestyle advice. The third and last step of the prevention program was treatment for patients with an increased risk for CMD with tailored lifestyle advice and/or medication.

Responders were defined as patients who completed the online or paper version of the RS within 3 months after receiving the invitation. The online or paper RS consisted of a seven item-questionnaire including age, gender, body mass index (BMI), waist circumference, family history of cardiovascular disease (CVD) and/or diabetes mellitus type II.

### 2.4 Characteristics of non-responders

The content of the non-response questionnaire was based on the literature (Groenenberg et al., 2016, Koopmans et al., 2012, Petter et al., 2015, Van der Meer et al., 2013) and previously developed questionnaires about participation in prevention programs (Nielen et al., 2009, Van der Meer et al., 2013).

The non-response questionnaire contained demographic characteristics (age, gender) and items on risk factors for CMD (smoking status, BMI, family history of type II diabetes mellitus and CVD, physical activity and alcohol consumption). Patients self-reported on weight and height, BMI was calculated afterwards. The risk factors obtained via the questionnaire were equal to the items in the RS. In addition the survey included questions about reasons for non-response, attitudes towards response-enhancing strategies and statements about CMD and screening.

Smoking status was defined as currently smoking (yes/no). BMI was calculated as weight/(height^2^) and a cut-off value of 25 kg/m^2^ was used to define overweight, a cut-off value of 30 kg/m^2^ was used to define obesity. Waist circumference was as defined as increased for females when measured 80 cm or over and for males 94 cm or over. A family history of CVD was defined as having first degree relatives with a cardiovascular event before the age of 65. Family history of DM was defined as having first degree relatives with diabetes mellitus type 2. The question about reasons for non-response had pre-set answer options, including an “other” option with a blanc field and non-responders could choose more than one answer. Answers on all statements about CMD and screening were formulated on a 5-point Likert scale, ranging from “totally agree” to “totally disagree”. For the data analysis ”totally agree” and ”agree” were combined in ”agree”, ”disagree” and ”totally disagree” in ”disagree”. The answers on the questions about attitudes towards response-enhancing strategies were formulated on a 3-point Likert scale, with “yes”, “maybe” and “no” as possible answers.

### 2.5 Statistical analysis

Descriptive analyses of all measurements were performed. To examine which factors are independently related to non-response, a multivariable multilevel logistic regression analysis was performed, using all variables to correct for possible confounding. In case of collinearity, the variable with the highest regression coefficient in aunivariate analysis was added to the model. Adjusted odds ratios and 95% confidence intervals were reported. Stata version 14 was used for all statistical analyses.

### 2.6 Ethical consideration

The INTEGRATE study, including this non-response analysis, was considered by the UMC Utrecht Institutional Review Board and exempted from full assessment under the Medical Research involving human subjects Act (Badenbroek et al., 2014).

## 3 Results

### 3.1 Response

**Fig. 1:**
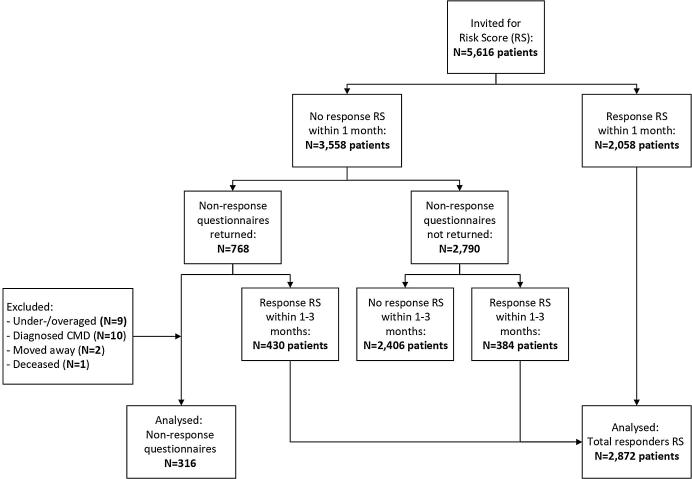
Flowchart of non-responders and responders risk score.

Of the 5616 patients that were approached 2058 (37%) completed the RS within the first month. One month after the initial invitation non-response questionnaires were sent to 3558 patients who had not completed the risk score by then. In addition 814 patients completed the risk score between 1 and 3 months’ time, adding up to a total response of N = 2872 (51%). A number of 768 non-response questionnaires were returned, a response rate of 22% (ranging from 13% to 33% between practices). We excluded 430 patients who had completed the RS after receiving the non-response questionnaire. Additional reasons for exclusion were patients with ages under 45 or over 70 (n = 9), patients reported to have a cardiometabolic disease (n = 10), patients who moved (n = 2) or deceased (n = 1). In total data from 316 non-responders were analyzed (see Fig. 1).

### 3.2 Characteristics of non-responders and responders

Characteristics of the non-responders and responders are listed in Table 1. Older patients seemed less likely to participate compared to younger patients, although this trend was not significant in the multivariate analysis. Non-responders were significantly more often smoker than responders (20 vs 15%, OR 0.67). The responders and non-responders did not differ according to gender and BMI. The waist circumference was too high in more than 72% of all subjects but no differences were seen between non-responders and responders. Because of the large amount of missing data on physical activity and alcohol consumption we were not able to add this variable to the analysis.

**Table 1:** Characteristics of non-responders and responders.

	Categories	N^a^	Non-responders (n = 316)	Responders (n = 2872)	Multivariate		
					OR	95% CI	p-value
Age	45–49 years	57	18%	23%			
	50–54 years	72	23%	24%	0.92	0.62–1.36	0.68
	55–59 years	61	19%	22%	0.96	0.64–1.44	0.85
	60–64 years	62	20%	16%	0.76	0.50–1.16	0.20
	65+ years	64	20%	16%	0.67	0.44–1.01	0.06
Gender	Male	154	49%	46%			
	Female	162	51%	54%	1.02	0.75–1.38	0.94
Body mass index	<25 kg/m^2^	129	48%	53%			
	25–30 kg/m^2^	111	41%	38%	0.97	0.72–1.29	0.81
	>30 kg/m^2^	28	11%	9%	0.86	0.54–1.38	0.54
Waist circumference	*Males*^b^*:*						
	<94 cm	31	33%	25%			
	≥94 cm	64	68%	75%			
	*Females*^b^*:*						
	<80 cm	14	14%	11%			
	≥80 cm	83	86%	89%			
	Increased				0.68	0.46–1.02	0.06
Family history of DM	Yes	49	18%	17%	0.99	0.71–1.40	0.97
Family history of CVD	Yes	82	30%	30%	1.05	0.79–1.40	0.74
Smoking	Yes	56	20%	15%	0.67	0.49–0.91	<0.01

DM, diabetes mellitus type 2, CVD, cardiovascular disease.

a Number of complete values for non-responders (complete data for responders).

b Males and females combined for multivariate analysis.

### 3.3 Reasons for non-response

The 316 patients reported 344 reasons for not responding (Table 2). The most reported reasons were ‘I forgot it’ (29%) and ‘I had no time’ (17%). In 21% of the reported reasons the patient stated having no need for a test, the patients felt healthy enough or didn’t want to know their risk. Of all reasons for non-response 14% was due to having been checked by a doctor recently. Study-specific causes including technical problems and privacy concerns accounted for 15% of the reasons for non-response.

**Table 2:** Reasons for non-response.

Reasons non-response (n = 344)	n	%
**Forgot/no time**	**159**	**46%**
I forgot	100	29%
I had no time	59	17%
**I have no need for a test**	**72**	**21%**
I feel healthy	21	6%
I don't want to know my risk	7	2%
I don't want to participate	23	7%
I already know what the results will be	21	6%
**Study-specific reasons**	**51**	**15%**
I have no access to internet	22	7%
I had technical problems with the website	11	3%
I have privacy concerns	12	3%
I didn't receive an invitation	6	2%
**Already checked by a doctor**	**48**	**14%**
I'm regularly checked by a doctor	30	9%
I'm recently checked by a doctor	18	5%
**Other**	**14**	**4%**

### 3.4 Statements

A large majority of the non-responders felt healthy (83%) and only 16% of the patients expected their own risk for CMD to be elevated (Table 3). Non-responders’ own estimation of being at increased risk ranged from 1% for chronic kidney diseases to 11% for CVD (data not shown). Almost three-quarters (73%) of the patients felt that they are able to keep themselves healthy, nevertheless a comparable part of the patients (75%) stated being willing to adjust their current lifestyle if that would be necessary for health reasons. Only one-third (34%) of the patients agreed with the statement that a GP should give advice about lifestyle.

**Table 3:** Statements of non-responders.

Statements:	Agree	No opinion	Disagree
I expect to have an elevated risk for cardiometabolic diseases	16%	33%	51%
I'm afraid for the results of the risk estimation	8%	22%	76%
I'm willing to adjust my lifestyle for my health	75%	15%	10%
I feel healthy	83%	8%	9%
I think the general practitioners should give advice about lifestyle	34%	29%	37%
I can take care of own health	73%	16%	11%
My family and friends find it important that I fill in the risk estimation	19%	57%	24%
I'm afraid others, like health insurance companies, find out the results of the risk estimation	24%	25%	51%

### 3.5 Attitude toward response-enhancing strategies

More than half of the non-responders would have performed or considered performing the risk score if the GP would ask him/her personally (Table 4). Almost half of the non-responders (45%) thought that making the risk score more visible by using advertisements in media could have convinced them to respond. An almost equal number (46%) thought that explaining more about CMD in the invitation letter could have positively influenced participation. Considerably less people were convinced about the positive effect of a meeting at the general practice (27%) or a reminder by telephone (22%). Thirty-three non-responders (37% of those who answered this question) would have considered filling in the risk score if it was available in their native language. However, only 4 of those 33 patients were migrants.

**Table 4:** Attitude of non-responders toward response-enhancing strategies.

Would you have considered completing the risk estimation in the following situations?	Yes	Maybe	No
If the general practitioners asked me to fill in the risk estimation personally	27%	31%	42%
If the risk estimation was more recognizable by use of advertisement	18%	27%	54%
If more explanation was given in the invitation letter	12%	34%	54%
If a meeting was originated at the general practice with help to complete it	8%	18%	74%
If I would be reminded by telephone	8%	14%	78%
If the risk estimation was available in my native language^a^	25%	12%	63%

FS, NdW, MN and MH contributed to the study concept and design. IB, DS, RK and the INTEGRATE team were involved in the acquisition of data. IB, AD, MN carried out the analysis and interpretation of data. IB, MN and AD participated in drafting the manuscript. FS, NdW, RK, DS and MH performed critical revision of the manuscript for important intellectual content. All authors have seen and approved the final version.

a 89 non-responders filled in this question.

### 3.6 Willingness to participate

Of all non-responders 73% seemed willing to participate, for they answered ‘yes’ or ‘maybe’ with one or more of the response-enhancing strategies. This group consisted mainly of patients who reported ‘forgot/no time’ and ‘study-specific reasons’ as reason for non-response. When comparing the answers on the statements of this specific group with all non-responders, they reported significantly more often that they feel healthy, that they are willing to adjust their lifestyle if necessary and that they can take care of their own health.

## 4 Discussion

### 4.1 Summary of results

In this non-response study we aimed at determining factors that influence response in a risk score for CMD, to be used as input for developing response-enhancing strategies. In multivariable multilevel regression analyses we found non-responders more often to be a smoker. Almost half of all reported reasons for not responding were either ‘forgotten it’ or ‘having no time’. Almost three quarter of the non-responders seemed willing to participate. Most non-responders felt healthy and expected their risk for CMD to be low, but also stated that they would be motivated to adjust their lifestyle to maintain healthy. A personal request from patients’ own GP is potentially the best method to enhance the response. Using advertisements and informative campaigns through media and more extensive information in the initial invitation are other methods that non-responders suggested.

### 4.2 Interpretation of results

Characteristics of non-responders in CMD prevention programs have shown variation in current literature. Lower response amongst younger age (Cochrane et al., 2013, Dalton et al., 2011, Hoebel et al., 2014, Klijs et al., 2012, Lambert et al., 2012, Weinehall et al., 1998) and smokers (Dalton et al., 2011, Klijs et al., 2012, Lang et al., 2016, Thorogood et al., 1993, Waller et al., 1990) was reported in most earlier studies in primary care. A possible explanation of the higher age trend among non-responders in our results is compatible with the often reported ‘worried well’ phenomenon, where responders to prevention programs tent be healthier but have higher levels of worry than non-responders (Barrett-connor and Austin, 1978). This could explain why the relatively healthy -younger- patients tend to seek more medical advice. Another possible explanation for the contrast in age of the non-responders in our study and the current literature is that a relatively older selection amongst the non-responders returned our non-response questionnaire.

Willingness to participate and willingness to adjust lifestyle was high amongst non-responders, which is in line with several earlier studies (Groenenberg et al., 2016, Petter et al., 2015, Wall and Teeland, 2004). Non-responders who are willing to participate have a favorable profile, they are willing to adjust their lifestyle and perceive control over staying healthy, which are both determinants for successful participation (de Waard et al., 2018, Groenenberg et al., 2016, Sonderlund et al., 2019). Most non-responders feel healthy and this may be one of the main reasons why it is so challenging to reach out to this group and getting them involved. The role of the GP in inviting these patients is yet to be specified further, for non-responders seem to value a personal approach. This is consistent with qualitative research that has been done amongst non-responders of the NHS health checks, where recommendations were made to emphasize personal relevance of participating (Burgess et al., 2015, Ellis et al., 2015). In the Netherlands a more personal approach through the GP has been proven successful and is implemented in the method of inviting women for cervical cancer screening since decades (Palm et al., 1993).

The non-responders in our study indicated that they desired to be informed better about CMD and risk factors. Other studies suggested more response in prevention programs could be achieved by increasing public awareness though media and giving more consideration to risk communication (Brannstrom and Lindblad, 1994, Burgess et al., 2015, Ellis et al., 2015, Wall and Teeland, 2004). This study adds that this line of thinking is also confirmed by the concerning target group, the non-responders, which to our knowledge has not been reported before.

The non-responders in our study surprisingly indicated that reminders by telephone would not persuade them, for this contrasts positive experiences with telephone reminders in the past (Godefrooij et al., 2012, Groenenberg et al., 2016).

### 4.3 Strengths and limitations

In this study we succeeded to gain relevant information from a substantial number of individuals that are usually hard to reach. Another strength of this non-response analysis is the integration in a large intervention study. This allows us to use the results as direct input for further exploration and testing response enhancing strategies in the same population. This is a unique design that creates great potential.

The most important limitation is the low response (22%) to the non-response questionnaire. However, considering this is response amongst non-responders, higher response rates may not be realistic. It is possible that there was a selective response of non-responders with a more positive attitude towards participation. This could mean that our conclusions concerning achievable response rates are somewhat overestimated. It is unclear if and to what extent this factor has biased the results of this study. Nonetheless, our results are comparable to Wall et al. (Wall and Teeland, 2004) who were able to get a response of 93% to their non-response questionnaire. Another limitation is that we didn’t measure willingness to participate directly but as derivative from other statements. The substitute measurements may not entirely reflect true willingness to participate and therefore could have biased our results.

## 5 Conclusions

High participation rates are crucial for successful prevention programs. So far, non-response in prevention programs in primary care has not been given sufficient attention. Our non-response analysis shows a clear message of potential for participation in prevention programs for CMD. Willingness to participate amongst non-responders is high and there are strategies we can use to reach them. Response enhancing strategies have been successful for other prevention programs in the past (Camilloni et al., 2013, Jepson et al., 2000). Persuasion of at least half the non-responders with the right methods seems a realistic goal. This means that with more time and energy we should be able to substantially boost response rates. A next logical step in this process is to test potential response enhancing strategies in research setting.

## 6 Author statement

FS, NdW, MN and MH contributed to the study concept and design. IB, DS, RK and the INTEGRATE team were involved in the acquisition of data. IB, AD, MN carried out the analysis and interpretation of data. IB, MN and AD participated in drafting the manuscript. FS, NdW, RK, DS and MH performed critical revision of the manuscript for important intellectual content. All authors have seen and approved the final version.

## Funding

This work was supported by ZonMW (The Netherlands Organization for Health Research and Development) under grant number 50-51515-98-192; Lekker Lang Leven (a collaboration of the Dutch Diabetes Research Foundation, the Dutch Heart Foundation and the Dutch Kidney Foundation) under grant number 2012.20.1595; and Innovatiefonds Zorgverzekeraars (Healthcare Insurance Innovation Fund) under grant number 2582.

## Declaration of Competing Interest

The authors declare that they have no known competing financial interests or personal relationships that could have appeared to influence the work reported in this paper.
